# Supplementary material for: The role of safety in modal choice and shift: A transport expert perspective in the state of Victoria (Australia)
Source: PLoS One. 2023 Apr 10;18(4):e0280949. doi: 10.1371/journal.pone.0280949 (PMC10085019; doi:10.1371/journal.pone.0280949)
Supplement: S1 File — (PDF) [file pone.0280949.s001.pdf]

The role of safety in modal choice and shift: A transport expert perspective in the state of Victoria (Australia)

Interview summary scripts

Interview with: Public Transport Victoria (PTV)

Date: 15/11/2017, Face-to-Face, 42 minutes

Q 1. What do you think are the major problems with the Victorian Transportation system at present?

Public Transport Planning

- Rapidly growing population
- More demand on transport
- Growing car dependency
- Proximity
- Problem with space, scatter
- Preferred modes car
- Pollution
- Hard for public transport to compete with car
- Public transport is poor in some area and people cant get easily
- Safety issues of people being killed on the roads
- Modal choices are rational
- The way the transport system is priced, roads are free and affordable

Q 2. What are the possible solutions under consideration?

Public Transport Planning

- Investment in infrastructure to support alternative mode of travel such trains and buses
- Make sure the infrastructure and services are thee to support public transport
- Make the other options more attractive and safer such segregated cycling
- Road use pricing
- Most people don’t see the external cost of their travel and the impact on the economy overall
- People might understand the implication of their decisions but the end they make their decision and if no body penalize them they will continue to do what they do

Interview summary scripts

Q 3. If we are looking for Modal Choice to improve safety, can you explain what is meant by Modal Choice as a potential strategy for improving road safety?

Public Transport Planning

- If you try to influence people choice from safety only it will be very weak strategy
- Because the other factors which influence modal choices will over this factor

Q 4. Do you have any thoughts or policies for using modal choice to improve road safety?

Public Transport Planning

- To make safer alternative more attractive to people such providing the infrastructure , the services make it more frequency
- Address personal security issues
- Make it more attractive
- Address the primary factors

Q 5. What do you believe are the factors that influence the way people choose their travel modes?

Public Transport Planning

- The cost of parking
- If you look to CBD data 70 % of trips to work b public transport Doclands 40 % by public transport but clayton is 5-10%
- Free parking to employees
- Public transport usually is slower that private vehicle
- If car is faster and parking is free it will be difficult for public transport to compete
- Congestion give opportunity to public transport as it can compete with it is own and segregated lanes
- Pricing , the cost of driving is quite low and people don't understand clearly the cost of their driving
- Have people really perceived the cost of their travel , people believe the cost of driving is cheaper that using public transport
- Travel time will be a massive issue

- Convenience
- Stops and transfer of public transport is barrier
- Travel time budget is related how far away you will accept a job
- Attractive some people see public transport attractive , they can drink and travel others see personal space in car is more attractive

Q 6. What role do you think safety plays in these choices?

Public Transport Planning

- Safety is not a primary drivers and has minimum role in modal choice,
- Safety could play barrier for some modes such cycling and walking
- There are a clear different between men and women in cycling especially
- Safety is a massive consideration for cycling only
- but personal safety or security plays major role especially for women and night time

Q 7. What do you see as your organization role in influencing people’s choice of mode of travel?

Public Transport Planning

- Our job is to provide attractive public transport service
- We need to do the best out of the fund we get so we need to target what we think will increase the use of the service such students central city workers major shopping centres the most possible market to shift
- Journey to work where parking is an issue is potential market
- Students are primary target due to low car ownership and income
- Establish more route that more frequent and direct
- Provide more time competitive public transport service

Q 8. Does your organization actively encourage people to choose safer modes of travel?

Public Transport Planning

- PTV try to address safety issues within their service such slip and fall , they organize campaigns for public such how to access with pram , how to park your pram

- Customer behaviour within public transport

Q 9. How – policies, practices, etc.?

Public Transport Planning

No answer

Q 10. Do you think that getting people to choose safer modes of travel would be an effective solution?

Public Transport Planning

- By definition yes but you need to come over the obstacles
- Shift people to cycling need to change the perspective of the risk

Q 11. How would modal choice fit into a safe system approach to road safety?

Public Transport Planning

- It does not fit it assume that everybody will continue the same way they travel
- However, a safer roads will provide safer environment for cycling and pedestrian
- Public transport has different approach

Q 12. How do you see the role of car sharing or ride sharing (GoGet,Flexidrive,Uber) on influencing travel choices?

Public Transport Planning

- Car sharing does facilitate lower car ownership and help in modal shift
- Ride sharing is talking away from buses and may increase the train users, it assist trains to get in to and from station. it is taking people away from feeder services .
- People are shifting from bus to Uber .
- Normally first and last mile should be solved with buses walking and cycling which does not required too much spaces for parking
- It is interesting to study the safety of uber and other taxi service against general travel

Interview summary scripts

Q 13. What do you believe is possible impact of disruptive technology in the way people choose to travel?

Public Transport Planning

- Mobility as services could have negative impact on safety
- Autonomous vehicles could have positive impact on safety for their promoted features but is going to work it is different
- Autonomous bus will increase the efficiency of bus service and can reduce the current risk and safety issue of using buses such fall and slip .
- Autonomous vehicle will cause massive congestion because of the number of such vehicles will be running on the roads

Q 14. Other thoughts or suggestions?

Public Transport Planning

No answer

The role of safety in modal choice and shift: A transport expert perspective in the state of Victoria (Australia)

Interview summary scripts

Interview with: Transport Accident Commission

Date: 20/11/2017, Telephone Interview, 50 minutes

Q 1. What do you think are the major problems with the Victorian Transportation system at present?

Transport Accident Commission

- Availability of options
- Lack of space to accommodate safety measures
- Integration
- Lack of safe options for active transport in the suburban areas
- Putting safety at the heart of the system in all geographical areas
- Safety is not a big factor in modal choice
- We are considering walking and cycling as transport modes but they are still not real options

Q 2. What are the possible solutions under consideration?

Transport Accident Commission

- Much more integration between modes
- Integration of the whole journey rather than transport mode
-

Q 3. If we are looking for Modal Choice to improve safety, can you explain what is meant by Modal Choice as a potential strategy for improving road safety?

Transport Accident Commission

- Public transport is safer than private driving, so if we move people to travel by public transport it will be safer but public transport could not be available for some people. Or people prefer to use other modes than public transport.
- Depending on the availability of options, people choose the safest mode

- We can provide more information for people about what modes are available and which is the safest mode for them to choose.
- Even within the same mode there are differences in safety and providing such information will help people how to make a safer choice.

Q 4. Do you have any thoughts or policies for using modal choice to improve road safety?

Transport Accident Commission

- We want to make active transport safer so we can encourage an increase in walking and cycling to achieve other health and well-being benefits.
- Providing alternatives for people and making them safe would be ideal.

Q 5. What do you believe are the factors that influence the way people choose their travel modes?

Transport Accident Commission

- How convenient they can travel
- Price, people are sensitive to price
- What are the other benefits than safety you can achieve from your choice, for example, cycling provides health and fitness benefits. They are looking at the whole package and components of benefits and each individual has a different response to how he perceived the benefits (elasticity)
- Where do they work and live
- Commitment during the journey to pick up or drop off kids for example.

Q 6. What role do you think safety plays in these choices?

Transport Accident Commission

- Safety is a major barrier to cycling. People responded that they don't cycle because they don't feel safe.
- I don't think choosing a mode because it is safe is part of people's decision-making. People have some level of risk acceptance.
- People don't have information about the relative risk between different modes. They might think motorcycling is risky but they don't know by how much if you compare it to other modes.

Q 7. What do you see as your organization role in influencing people's choice of mode of travel?

- Does your organization actively encourage people to choose safer modes of travel?

Q 8. How – policies, practices, etc.?

Transport Accident Commission

- We don't influence which mode to choose, but we make sure for whatever mode people choose we provide the information to do your choice as safely as possible
- To supply the requirements to make safe options available for people's choice and then encourage them to use these safe options

Q 9. Do you think that getting people to choose safer modes of travel would be an effective solution?

Transport Accident Commission

- Safety by itself would not be an effective factor to influence people's choices. It has to be part of a package of factors that attract people to the safe options.

Interview summary scripts

Q 10.How would modal choice fit into a safe system approach to road safety?

Transport Accident Commission

- To encourage the use of safer ways of travel, for example, to encourage cycling on separate lanes rather than mixed lanes with general traffic sharing the road with other bigger vehicles.

Q 11.How do you see the role of car sharing or ride sharing (GoGet,Flexidrive,Uber) on influencing travel choices?

- Transport Accident Commission
- It is more shifting people from private driving but not from public transport
- It is not going to replace public transport
- It will have some safety benefits from shifting people from a car driving to being car passengers and the car that are used in car-sharing companies are at a better safety level than some private cars

Q 12.What do you believe is possible impact of disruptive technology in the way people choose to travel?

Transport Accident Commission

- Technology is part of road safety improvements, but it is not clear how long it will take

Q 13.Other thoughts or suggestions?

Transport Accident Commission

The role of safety in modal choice and shift: A transport expert perspective in the state of Victoria (Australia)

Interview summary scripts

Interview with: Bus Association Victoria

Date: 22/11/2017, Face-to-Face, 58 minutes

Q 1. What do you think are the major problems with the Victorian Transportation system at present?

Bus Association Victoria

- We don’t have any plan , planning strategy does not have transport part
- We don’t have any long term goal to achieve , all what we have is 4 years strategy which is only incremental approach
- The absent of the grand master transport plan
- Land planning and transport planning should be integrated
- They do this integration only selectively occasionally although there is an obligation for land use and transport plans to be integrated
- The legislation says the government may do this but not they should do this
- We are pushing the city to wider area and more suburbans without prober transport infrastructure and no provision for public transport buses walking or cycling tracks
- We are developing social inequity between the city and outer suburbans because the availability of transport options on the outer suburbans are very limited
- The urban sprawls has built this dependency on cars and you don’t have good service for other options
- Less desirable of destinations in the outer suburbans
- The absence of multigenerational transport plan
- The political system of 4 years election does not allow for long-term commitment of transport projects
- The solution is to agree on a long-term land use transport master plan
- Lag mind set , growing without thinking how we are going to serve this growing
- It reactive policies destroying the original nice grid system

Interview summary scripts

Q 2. What are the possible solutions under consideration?

Bus Association Victoria

- Reframe Melbourne with transport options better for our economies better for our environment and reduce the carbon footprint
- At the current we are car dependency which is very bad for our system, cars are not produced locally anymore so the jobs are spilled over the country
- Cars present affluent which is not god for our social standing , if we want to increase social capital you better catch public transport
- If you time concern which is resulted from strong economic
- Car is very bad for environment
- we need to reframe the city to achieve better economy social and environment and private car cannot achieve this
- people talk about sharing economy but actually public transport is the sharing economy

Q 3. If we are looking for Modal Choice to improve safety, can you explain what is meant by Modal Choice as a potential strategy for improving road safety?

Bus Association Victoria

- It sounds like people should be educated to what level of risk is associated with different modes of transport so the level of safety can influence commuter choices
- I don't think will influence their decision but it is good to know
- Also information is not available
- The improvement of safety can come under the economic benefits

Q 4. Do you have any thoughts or policies for using modal choice to improve road safety?

Bus Association Victoria

- Any initiatives to get people out of their car to public and active transport
- We need to make car as unattractive options with road pricing regimes to charge people who choose car for their personal reasons but you take this money and invest it directly in public and active transport so ideally we will have less cars and better public transport service

Interview summary scripts

- Parking pricing is limited but we need a pricing regime where the beneficially pay directly (direct tax used directly to public transport) double intensives make care less attractive and make public transport more attractive
- Hypothecation the money come from private vehicle and go to public transport
- Change land use approach clustering the state rather city concertation

Q 5. What do you believe are the factors that influence the way people choose their travel modes?

Bus Association Victoria

- The purpose of the trip
- How much do you have time availability

Q 6. What role do you think safety plays in these choices?

Bus Association Victoria

- Safety is a factor but because we all was told that safety is a concern when travelling at night in public transport – personal safety – is the safety concern from individual perception
- Safety is very low down the ladder .
- People some times think about it for occasion trips such travelling on charismas using car or flight
- The level of safety concern change based on different modes , for public transport people who are not using public transport are concerned about their personal safety
- People who drive do not think that they are going to have accidents but it happens and it could be not your fault people do not think about that
- The extend of thinking about safety change depends on what mode they are taking
- Sometimes it is barrier more for public transport and less for cars
- Less knowledge about the safety of different modes

Interview summary scripts

Q 7. What do you see as your organization role in influencing people's choice of mode of travel?

Bus Association Victoria

- Our role is to influence the government to introduce initiatives that improve the public safety.
- Is to work with government to help them improve the service
- Advocacy group make the public transport service safer and better

Q 8. Does your organization actively encourage people to choose safer modes of travel?

Bus Association Victoria

- We as industry representative is to influence government to influence people to get out of their cars and use public transport which is much safer and better for economy social and environment

Q 9. How – policies, practices, etc.?

Bus Association Victoria

- Develop one in five campaign to encourage people to use public transport one day a week
- Bick racks on buses
- Undertake bus safety week to the public

Q 10. Do you think that getting people to choose safer modes of travel would be an effective solution?

Bus Association Victoria

- Yes but there is a big job ahead to make a safety is key factor , it needs behaviour change which needs time but it is possible , people need to know the risk
- It will be effective for some and not for others based on individual case but even if few did this will be beneficial
- Our transport system is a safe system in general which make other factors more important
- Every transport mode is safe

Interview summary scripts

Q 11. How would modal choice fit into a safe system approach to road safety?

Bus Association Victoria

- I am not familiar about it
- But transport does not mean roads

Q 12. How do you see the role of car sharing or ride sharing (GoGet,Flexidrive,Uber) on influencing travel choices?

Bus Association Victoria

- They will come and go
- Uber are taking market share from public transport especially from buses because it is affordable

Q 13. What do you believe is possible impact of disruptive technology in the way people choose to travel?

Bus Association Victoria

- This will happen but it will take long time
- Impact on other modes will be limited

Q 14. Other thoughts or suggestions?

Bus Association Victoria

No answer

The role of safety in modal choice and shift: A transport expert perspective in the state of Victoria (Australia)

Interview summary scripts

Interview with: Transport Safety Victoria

Date: 29/11/2017, Face-to-Face, 50 minutes

Q 1. What do you think are the major problems with the Victorian Transportation system at present?

Transport Safety Victoria

- Integration of transport services especially bus service
- Bus incident related to Slip, trip or fall especially for elderly people

Q 1. What are the possible solutions under consideration?

Transport Safety Victoria

- Reduce the number of Slip, trip or fall
- Stakeholders engagement including bus drivers, bus users and other road users such car drivers
- Behavioral change such young users attitude to stand for elderly people and bus drivers to drive more carefully and gently , other road users to respect bus movements and consider people inside the buses
- Potential solutions to infrastructure such the internal design of the buses
- Enhancement to physical on and off the bus

Q 2. If we are looking for Modal Choice to improve safety, can you explain what is meant by Modal Choice as a potential strategy for improving road safety?

Transport Safety Victoria

- It is not clear if modal choice can be used as safety strategy because the expectation from transport users that all modes are safe and the transport system is provided by the government as safe system.
- Transport users assume all modes are safe

Q 3. Do you have any thoughts or policies for using modal choice to improve road safety?

Transport Safety Victoria

No answer

Interview summary scripts

Q 4. What do you believe are the factors that influence the way people choose their travel modes?

Transport Safety Victoria

- Convenience, comfort, price, proximity

Q 5. What role do you think safety plays in these choices?

Transport Safety Victoria

- No role Safety has minimum role in modal choice , but personal safety or security plays major role

Q 6. What do you see as your organization role in influencing people's choice of mode of travel?

Transport Safety Victoria

- Provide confident on using bus service , we don't influence modal choice but we influence the way people use our modes

Q 7. Does your organization actively encourage people to choose safer modes of travel?

Transport Safety Victoria

- We encourage people to be safe when using the mode they choose to use

Q 8. How – policies, practices, etc.?

Transport Safety Victoria

- Bus safety ACT ADRS DIRA
- Stakeholders engagement including bus drivers, bus users and other road users such car drivers
- Behavioral change such young users attitude to stand for elderly people and bus drivers to drive more carefully and gently , other road users to respect bus movements and consider people inside the buses
- Potential solutions to infrastructure such the internal design of the buses
- Enhancement to physical on and off the bus

Interview summary scripts

Q 9. Do you think that getting people to choose safer modes of travel would be an effective solution?

Transport Safety Victoria

- No it Is not strong approach since people are assuming the current system is safe
- Mobility need drive the safety

Q 10. How would modal choice fit into a safe system approach to road safety?

Transport Safety Victoria

- Mobility need drive the safety
- Bus operation follow systematic approach such 5 why's
- No link between road safe system and bus safety system

Q 11. How do you see the role of car sharing or ride sharing (GoGet,Flexidrive,Uber) on influencing travel choices?

Transport Safety Victoria

- Give more choices which detracted bus users and less people will use buses
- Give ore convenience than using buses

Q 12. What do you believe is possible impact of disruptive technology in the way people choose to travel?

Transport Safety Victoria

- Choices move toward convenience than safety
- It will take long time because changes in regulation take time

Q 13. Other thoughts or suggestions?

Transport Safety Victoria

No answer

Interview summary scripts

Interview with: Royal Automobile Club of Victoria (RACV)

Date: 06/12/2017, Face-to-Face, 35 minutes

Q 1. What do you think are the major problems with the Victorian Transportation system at present?

Royal Automobile Club of Victoria (RACV)

- Disjointed system
- Not integrated
- It relies on people taking transport to the city
- It is city centric
- Modes are not integrated it relies on chances
- Relying on people travel to the city rather than a cross functionality
- Transportation system is concentrated in the city rather than give you a cross town
- It is also an issue with roads , there is only two main roads to the city and you need to cross the CBD to be able to reach from one side to the other side

Q 2. What are the possible solutions under consideration?

Royal Automobile Club of Victoria (RACV)

- Do a link roads
- Rail infrastructure

Q 3. If we are looking for Modal Choice to improve safety, can you explain what is meant by Modal Choice as a potential strategy for improving road safety?

Royal Automobile Club of Victoria (RACV)

- The solution is not one or another it is whole system
- Modal shift should consider people to use all mode of transport
- It is not about building more rail routes only or getting people out of their cars
- Transport options should be more in cooperated greater connections smart connections
- Essentially if you move people out of their cars you will reduce their risk that is one method

Interview summary scripts

Q 4. Do you have any thoughts or policies for using modal choice to improve road safety?

Royal Automobile Club of Victoria (RACV)

- Publicize the choices that are available , people are not aware of the available options
- Improve the safety of people when they change mode to another such pedestrian and cycling
- The downsize of it is to make the transport hub where people change ode to another is more friendly and safe particularly for pedestrian and cyclist
- More integration

Q 5. What do you believe are the factors that influence the way people choose their travel modes?

Royal Automobile Club of Victoria (RACV)

- The availability of the modes and transport options
- The cost
- The time
- The security

Q 6. What role do you think safety plays in these choices?

Royal Automobile Club of Victoria (RACV)

- Depends on the mode safety as personal security
- Not crashing train is safer if cycling this is the first thing to think about it
- For vehicles safety is the in the middle and it is important for the purchasing choice
- For cycling safety is a major barrier

Q 7. What do you see as your organization role in influencing people's choice of mode of travel?

Royal Automobile Club of Victoria (RACV)

- We are information providers ,

- Inform public the end users what options are available with advantages and disadvantages
- Lobby group to influence government decisions
- Public campaigns
- Provide information to make the right decision rather than influence everybody to choose particular mode
- There is no one right solution for everybody

Q 8. Does your organization actively encourage people to choose safer modes of travel?

Royal Automobile Club of Victoria (RACV)

- We encourage people to be safe while using any mode of transport , there should be no safe mode and other risky mode
- Safer car
- Safer road users
- Safer cycling
- Safer roads

Q 9. How – policies, practices, etc.?

Royal Automobile Club of Victoria (RACV)

No answer

Q 10. Do you think that getting people to choose safer modes of travel would be an effective solution?

Royal Automobile Club of Victoria (RACV)

- NO if you choosing between mode and another it is not realistic to bas e your decision on safety only but if you are choosing within the same mode , then it is definitely factor such choosing to purchase different model of cars and the type of equipment
- We are in the business of telling people how they can be safe in a particular mode but not telling people to choose and particular mode
- When I choose between car and train , my choice is govern by time cost and availability not safety but I will make sure that my choice is safe

Interview summary scripts

Q 11. How would modal choice fit into a safe system approach to road safety?

Royal Automobile Club of Victoria (RACV)

- It can be related to when you shift modes it is important to insure the safety.
- Other modes than car such train and tram are based on the safety but it is different from the road safety system
- Buses, walking and cycling would be subject and part of the safe system approach

Q 12. How do you see the role of car sharing or ride sharing (GoGet,Flexidrive,Uber) on influencing travel choices?

Royal Automobile Club of Victoria (RACV)

- Maybe for short journey only
- These business models are on lost currently
- Uber could assist the metro transport system in the first and last mile

Q 13. What do you believe is possible impact of disruptive technology in the way people choose to travel?

Royal Automobile Club of Victoria (RACV)

- People will still needs to buy car for different reasons and have them available
- This future is taking the role of taxi and the change is only taking the drivers out but not replacing the other cars
- It is not everyday journey , it is ad hock
- It tends to be casual and emergency
- It will take long time for law and regulations but the vehicles itself already in the market

Q 14. Other thoughts or suggestions?

Royal Automobile Club of Victoria (RACV)

No answer

Interview summary scripts

Interview with: POPCAR Car Sharing

Date: 04/12/2017, Video call, 40 minutes

Q 15. What do you think are the major problems with the Victorian Transportation system at present?

Car Share Expert

- Population growth without transport infrastructure
- Car ownership
- Congestion
- Ability to move smoothly from work to home

Q 16. What are the possible solutions under consideration?

Car Share Expert

- Reduce the private car trips on the roads and reduce the pressure on the transport network

Q 17. If we are looking for Modal Choice to improve safety, can you explain what is meant by Modal Choice as a potential strategy for improving road safety?

Car Share Expert

- Change in the manufactory technology of the vehicles
- Car share will reduce time of travel which improve safety

Q 18. Do you have any thoughts or policies for using modal choice to improve road safety?

Car Share Expert

- Car sharing

Q 19. What do you believe are the factors that influence the way people choose their travel modes?

Car Share Expert

- It works financially for them
- Convenient
- Easy accessible

- Time to get in and out

Q 20. What role do you think safety plays in these choices?

Car Share Expert

- We have not really measure it
- Our car is new and the age of our fleet is new than typical private vehicles fleet , so it is somehow safe in term of the quality ,technology and features of the vehicles.

Q 21. What do you see as your organization role in influencing people's choice of mode of travel?

Car Share Expert

- We are providing alternative to driving private car to reduce congestion and take some pressure from the infrastructure
- It is win - win for everybody it is win for the people who use it and win for the community who does not use it in reducing the amount of vehicles win for the environment

Q 22. Does your organization actively encourage people to choose safer modes of travel?

Car Share Expert

- We maintain our fleet to be safe for use by our customers
- We choose our fleet with a good safety features
- We provide safe vehicles
- Safety a key for people when they do their purchasing decision for new vehicle and the same can be applied when they choose our service

Q 23. How – policies, practices, etc.?

Car Share Expert

No answer

Interview summary scripts

Q 24. Do you think that getting people to choose safer modes of travel would be an effective solution?

Car Share Expert

No answer

Q 25. How would modal choice fit into a safe system approach to road safety?

Car Share Expert

- Modal choice fit in term of choosing the safe vehicle and car sharing provide safe vehicles for use
- We have policy to remove any bad drivers form our car sharing community
- We have very low level of traffic incidents

Q 26. How do you see the role of car sharing or ride sharing (GoGet,Flexidrive,Uber) on influencing travel choices?

Car Share Expert

- I think we provide alternative transport options to private vehicle
- We give more options
- We increase the education and awareness of people about alternatives
- The more we have people using car sharing the more the alternatives will be accepted
- We support public transport and fit within the service to integrate with them
- We don't believe that we replace public transport we do not provide the full journey we just take part of any journey and support the main journey mode , it is part of the people travel behaviour
- We have some examples of car sharing companies that integrate with rail system the large distance done by train and the last part done by car sharing

Q 27. What do you believe is possible impact of disruptive technology in the way people choose to travel?

Car Share Expert

- Vehicles are going to change over the coming years especially electrical vehicles and it just need this type of vehicles to be price competitive
- To be adopted widely

- This will be good for the car sharing scheme

Q 28. Other thoughts or suggestions?

Car Share Expert

No answer

The role of safety in modal choice and shift: A transport expert perspective in the state of Victoria (Australia)

Interview summary scripts

Interview with: Transport for Victoria

Date: 30/10/2018, Video Call58 minutes

Q 1. What do you think are the major problems with the Victorian Transportation system at present?

Transport for Victoria

- Challenges in Applying the Transport Integration Act outcomes: Integration, safety and well being
- Population growth putting stress on transport infrastructure
- Shift from public transport (efficient mode) to better mobility as service options(cars)
- Challenges in Sustainability, transport impact on the environment and the climate change

Q 2. What are the possible solutions under consideration?

Transport for Victoria

- Expenditure in major infrastructure projects by expanding heavy and light rails to move people to public transport
- The use of electricity in rail and tram network to reduce the environment and emissions impact
- Road safety improvements
- Try to understand mobility as service needs such regularity issues

Q 3. If we are looking for Modal Choice to improve safety, can you explain what is meant by Modal Choice as a potential strategy for improving road safety?

Transport for Victoria

- There is gap between towards zero and modal choice especially the efficient choice such public transport is a safe mode
- Disruption management, the government has significant infrastructure expansion which has positive impact on the long term but it could have negative impact during the construction
- Active transport Victoria is trying to promote walk cycling for health purpose but not for the safety

Interview summary scripts

Q 4. Do you have any thoughts or policies for using modal choice to improve road safety?

Transport for Victoria

No answer

Q 5. What do you believe are the factors that influence the way people choose their travel modes?

Transport for Victoria

- Convenience, this why people in the sub areas prefer cars
- Price, this is the concern of shifting people from public transport to new mobility as service
- Environment is not significant consideration for many people

Q 6. What role do you think safety plays in these choices?

Transport for Victoria

- Very little impact for day to day because our transport system is safe
- It is clearer when people take the decision to purchase new car

Q 7. What do you see as your organization role in influencing people's choice of mode of travel?

Transport for Victoria

- Informing the public and the industry how performance and operation should be but we don't have major role in directly influence people choice

Q 8. Does your organization actively encourage people to choose safer modes of travel?

Transport for Victoria

- Not relay direct, we need to be carful of our massages in how we influence people choices

- We don't directly promote specific mode from safety perspective for different reasons such : contract issues not to impact specific businesses

Q 9. How – policies, practices, etc.?

Transport for Victoria

Q 10. Do you think that getting people to choose safer modes of travel would be an effective solution?

Transport for Victoria

- Yes, but it should be part of border picture
- We want improve safety across all modes

Q 11. How would modal choice fit into a safe system approach to road safety?

Transport for Victoria

- It is focus on roads only but not the full transport system

Q 12. How do you see the role of car sharing or ride sharing (GoGet,Flexidrive,Uber) on influencing travel choices?

Transport for Victoria

- Challenges in the infrastructure such providing more drop on and drop off points
- We don't know if there are any safety implications
- It depends on geographic and demographic location and their coverage
- Social exclusion and equity if car sharing choose not to serve specific areas
- They have impact but not consistent across all locations
- We don't have data on the risk differences between private and commercial vehicles
- Commercial Passenger Vehicles Victoria is a new entity will have more insight about the safety of new commercial mobility options.

Interview summary scripts

Q 13. What do you believe is possible impact of disruptive technology in the way people choose to travel?

Transport for Victoria

- Performance-Based Regulation and Regulatory Regimes
- Disruptive technology will have impact, but we don’t know exactly to what extend

Q 14. Other thoughts or suggestions?

Transport for Victoria

This is a personal experience views
