# Supplementary material for: The role of safety in modal choice and shift: A transport expert perspective in the state of Victoria (Australia)
Source: PLoS One. 2023 Apr 10;18(4):e0280949. doi: 10.1371/journal.pone.0280949 (PMC10085019; doi:10.1371/journal.pone.0280949)
Supplement: Attachment — Submitted filename: Reviewer Comments & Rebuttal.docx [file pone.0280949.s002.docx]

**Subject: Revision required**

**Dear Editor,**

We are sending a revised manuscript entitled “The role of safety in modal choice and shift: A transport expert perspective in the state of Victoria (Australia)” that replace the previous title “EXPERT PERSPECTIVES OF MODAL SHIFT AND SAFETY IN THE STATE OF VICTORIA (AUSTRALIA)”. We have addressed the academic editor and reviewers in the revised manuscript and below are our responses to the comments.

| **General Comments** | **Response** |
| --- | --- |
| 1.Please ensure that your manuscript meets PLOS ONE's style requirements, including those for file naming. The PLOS ONE style templates can be found at  https://journals.plos.org/plosone/s/file?id=wjVg/PLOSOne_formatting_sample_main_body.pdf and  https://journals.plos.org/plosone/s/file?id=ba62/PLOSOne_formatting_sample_title_authors_affiliations.pdf | We followed PLOS ONE's style requirements in the revised manuscript. |
| 2. In your Data Availability statement, you have not specified where the minimal data set underlying the results described in your manuscript can be found. PLOS defines a study's minimal data set as the underlying data used to reach the conclusions drawn in the manuscript and any additional data required to replicate the reported study findings in their entirety. All PLOS journals require that the minimal data set be made fully available. For more information about our data policy, please see http://journals.plos.org/plosone/s/data-availability | Interview manuscripts will be attached |
| Upon re-submitting your revised manuscript, please upload your study’s minimal underlying data set as either Supporting Information files or to a stable, public repository and include the relevant URLs, DOIs, or accession numbers within your revised cover letter. For a list of acceptable repositories, please see http://journals.plos.org/plosone/s/data-availability#loc-recommended-repositories. Any potentially identifying patient information must be fully anonymized. | **Interview manuscripts will be attached** |
| Important: If there are ethical or legal restrictions to sharing your data publicly, please explain these restrictions in detail. Please see our guidelines for more information on what we consider unacceptable restrictions to publicly sharing data: http://journals.plos.org/plosone/s/data-availability#loc-unacceptable-data-access-restrictions. Note that it is not acceptable for the authors to be the sole named individuals responsible for ensuring data access. | Interview manuscripts will be attached |
|  |  |

| **Reviewer 1**  This paper addresses an interesting issue (or set of them) through a set of semi-structured interviews applied to experts in the field. There are some comments that need your attention, as you will see below: | | |
| --- | --- | --- |
| Comment |  | Response to Reviewer |
|  |  |  |
| - Abstract could be structured and clearer, therefore. |  | The abstract was revised with structure subtitles and some rephrasing. |
| - The paper is written in a reader friendly way. However, many typos are spread throughout the manuscript. |  | The paper was revised and corrected |
| Introduction is not really convincing, as literature review is weak and literature on behavioral contributors to usar safety seems absent from this paper, even though good points in this regard are made in the results. |  | The introduction is revised, and a new section added about modal choice (travel behaviour) impact on road safety |
| *The methodological approach is superfluous. Qualitative research does not mean "technically poor" or "less rigorous" research. Please elaborate better on the methods used and the data analysis strategy.* On the other hand, the results are more organized an present acceptable ledvels of detail, even in absence of the methodological setting of the study in the previous section. |  | Section 2 includes the method it includes sub summary of the method steps in participant selection and the thematic analysis approach. The method section was revised to provide more clarity. |
| - Discussion does not really discuss key findings, but re-describes and paraphrases most of them. This needs strong revisions from the authors. |  | The discussion section was revised and reproduced |
| One of my main concerns is the lack of adequacy of the conclusions. As this is a very limited study in terms of coverage and data, my feeling is that what the authors conclude exceeds the actual scope of the data. Therefore, it would be suggestible to carefully revise each one of the conclusive statements, reshaping them if needed, in view of the key limitations of the study.  For instance, authors did well saying that their results "highlight" some expert perceptions. However, three lines below the statement is "data showed public transport modes have relatively lower risk of being seriously injured in road crash"... and this was not data directly related to your study, but it looks like anyway. |  | The conclusion section was revised |
| Line 437: This sentence is incomplete. |  | This is reproduced now |
| **Reviewer #2** |  |  |
| The research aimed to analyse the role of modal shift of road users in improving road safety through semi-structured interviews with experts. This is an interesting topic with important practical applications. However, I consider that some modifications should be made to the manuscript before it is considered for publication. |  | The manuscript has been revised and modified |
| consider that some modifications should be made to the manuscript before it is considered for publication.  The introduction should be developed further, setting out in detail the factors involved in modal choice and the implications for mobility and road safety. For example, evidence suggests that perceived safety may be a variable influencing route selection or the choice of a particular mode of transport for travel (e.g. doi: https://doi.org/10.3389/frsc.2020.00042 and https://doi.org/10.1016/j.tranpol.2012.09.009). |  | The issue of perceived safety as a factor in modal choice was highlighted in the results and discussions where it might play a barrier to choosing cycling as it is perceived as a high-risk mode. It was also captured in the results and discussions around older people’s perception of risk from other public transport users’ behaviour which might influence their choice to use public transport.  Thanks for the two references, and they were added to the discussion section. |
| Additionally, I recommend including the main hypotheses of the study together with the research objectives. |  | Thanks, it was added to the study objectives |
| The methodology and results are very comprehensive. And the discussion is adequate, having contrasted the results with similar research to explain and contextualise the data obtained. |  | Thanks, the manuscript was revised with further improvements |
| I only suggest including measures that can enhance modal shift based on the results and scientific evidence |  | This is beyond the scope of this study as it was only meant to explore if safety can encourage modal shift. However, we touched on that in the conclusion: “Working in parallel by addressing those factors and increasing the awareness of the different risks associated with different modes could potentially encourage the choice of more desirable safe modes” |
| The development of specific communication campaigns can be an effective tool for this purpose, especially if they are presented together with other complementary measures (e.g. doi: (https://doi.org/10.3390/safety7040066 and https://doi.org/10.5114/jhi.2016.61421) |  | Thanks, communication and information availability were added to the discussion and the two references were added |
| **Reviewer #3:** | | |
| The author failed to illustrate the conclusions with solid data and rigrous logitistics. The sample size of participants of interview is too small to obtain reliable conclusions. The author should expand the sample number and discuss the representativity of the samples. |  | The manuscript and conclusions were revised to better illustrate the study.  The sample size is enough as suggested by previous qualitative research, and this was discussed in the method section and supported by the previous literature. |
| Ambiguous or uncertain words are used in the results and conclusion parts, such as "somehow", "something" and so on.  The author should use more plots to directly show the results. For example, the percentages of each point of view. |  | The manuscript and conclusions were revised to better illustrate the study.  We don’t think using percentages for this qualitative study is necessary as the thematic analysis uses saturation as an approach to describe the bulk of ideas. |

We hope that the manuscript meets the journal requirements now and we are looking forward to receiving a favourable response from you regarding the acceptance of our manuscript.

Kind regards.,

Mohammad Nabil Ibrahim
